# Purpura pigmentosa progressiva unter Immuncheckpoint-Inhibitor-Therapie mit Pembrolizumab: Ein Fallbericht

**DOI:** 10.1007/s00105-026-05657-7

**Published:** 2026-03-12

**Authors:** Annia Cherrez-Eschrich, Amrei Dilling, Kamran Ghoreschi, Alexander Nast

**Affiliations:** Klinik für Dermatologie, Allergologie und Venerologie, Charité – Universitätsmedizin Berlin, corporate member of Freie Universität Berlin, Humboldt-Universität zu Berlin, Berlin, Deutschland

**Keywords:** Onkologische Therapie, Kutane Nebenwirkungen, Makulae, Histologie, Hautbiopsie, Antineoplastic protocols, Cutaneous adverse events, Macules, Histology, Skin biopsy

## Abstract

Immuncheckpointinhibitoren sind heute fester Bestandteil der onkologischen Therapie, und kutane Nebenwirkungen sind häufig. Wir berichten über eine 40-jährige Patientin, bei der im zeitlichen Zusammenhang mit Pembrolizumab eine Purpura pigmentosa progressiva (PPP) auftrat. In der Kasuistik wird PPP als eine seltene potenzielle Nebenwirkung von Immuncheckpointinhibitoren beschrieben.

## Abstract (en)

Immune checkpoint inhibitors are now an integral part of cancer treatment, and cutaneous side effects are common. We report the case of a 40-year-old woman who developed purpura pigmentosa progressiva (PPP) in temporal association with pembrolizumab. This case report describes PPP as a rare potential adverse effect of immune checkpoint inhibitors.

## Fallbeschreibung

Wir berichten über das Auftreten sowie den weiteren Verlauf einer Purpura pigmentosa progressiva (PPP) bei einer 40-jährigen Patientin unter einer Immuncheckpointinhibition mit Pembrolizumab.

Die Patientin befand sich wegen eines erstmaligen Rezidivs eines serösen Low-grade-Ovarialkarzinoms (Erstdiagnose 02/2019) seit Oktober 2024 in Behandlung im Rahmen einer klinischen Studie. Die Therapie umfasste zunächst eine Kombination aus Pembrolizumab (alle 3 Wochen), Carboplatin und Paclitaxel. Die Chemotherapie wurde regulär zum Februar 2025 abgeschlossen und Pembrolizumab als Monotherapie fortgesetzt.

Im Mai 2025 erfolgte die Erstvorstellung in unserer Abteilung. Die Patientin berichtete über das Auftreten von nicht juckenden, rötlich-braunen Makulae an den oberen und unteren Extremitäten, die zu diesem Zeitpunkt seit etwa 3 Wochen bestanden. Die Hautveränderungen traten somit ca. 5 Monate nach Therapiebeginn auf.

Es bestand keine relevante dermatologische Vorgeschichte, auch andere internistische oder systemische Grunderkrankungen waren nicht bekannt. Begleitmedikation war lediglich Escitalopram, das sie bereits seit 9 Jahren einnimmt. Nikotinkonsum oder systemische Infektionen wurden verneint. Aufgrund der Hautveränderungen wurde Pembrolizumab zunächst pausiert.

**Abb. 1:**
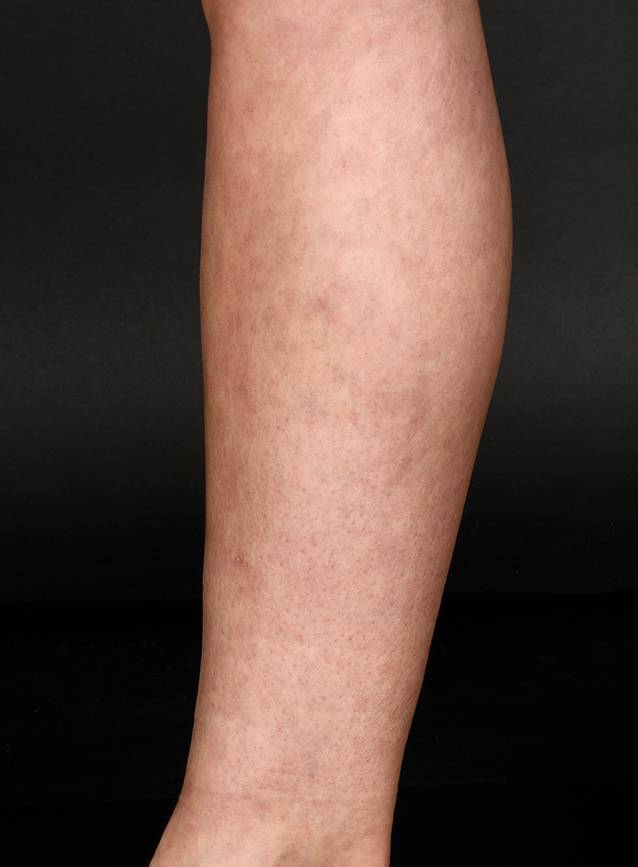
Klinischer Befund: rötlich-braune Makulae am linken Unterschenkel

Die klinische Untersuchung ergab symmetrisch verteilte, unscharf begrenzte, rotbraune Makulae an Armen und Unterschenkeln ohne Schuppung, Konfluenz oder Palpabilität (Abb. 1 und 2). Unter Glasspateldruck kam es zu keiner Veränderung des Befundes. Die Läsionen waren symptomlos; insbesondere bestand kein Juckreiz oder Schmerz. Schleimhäute waren unauffällig.

**Abb. 2:**
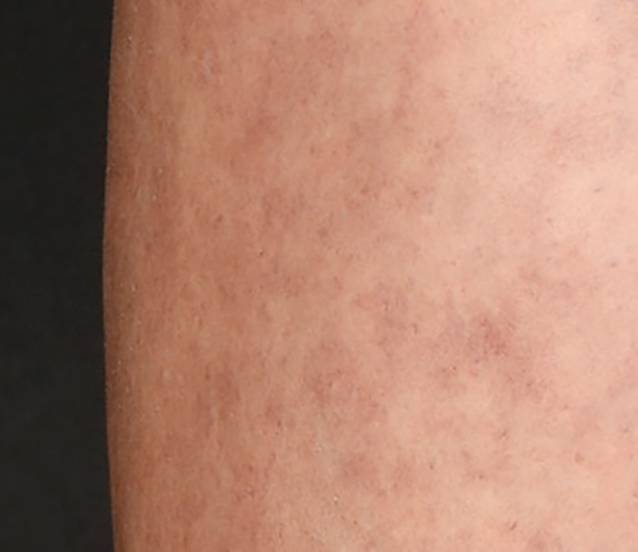
Detailansicht klinischer Befund

**Abb. 3:**
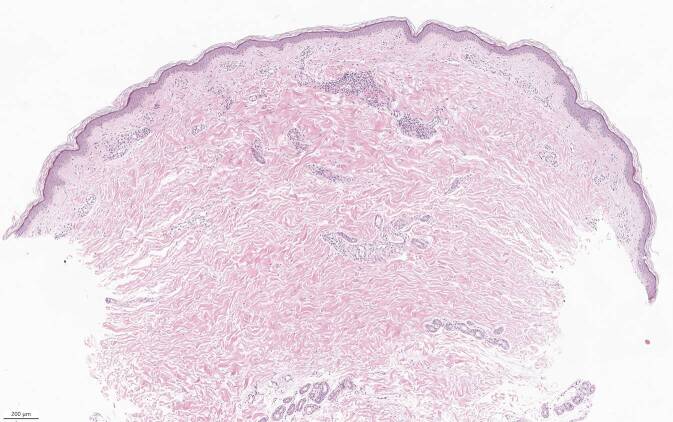
Stanzexzisat der Haut mit einem oberflächlich perivaskulär betonten lymphozytären Infiltrat (HE-Färbung)

Die klinische Verdachtsdiagnose einer Purpura pigmentosa progressiva (PPP) wurde durch die histopathologische Untersuchung einer Hautbiopsie bestätigt. Diese zeigte ein oberflächlich perivaskulär betontes lymphozytäres Infiltrat mit diskreten Erythrozytenextravasaten – ein histologisches Reaktionsmuster, das im frühen Stadium einer PPP zu beobachten ist (Abb. 3 und 4).

**Abb. 4:**
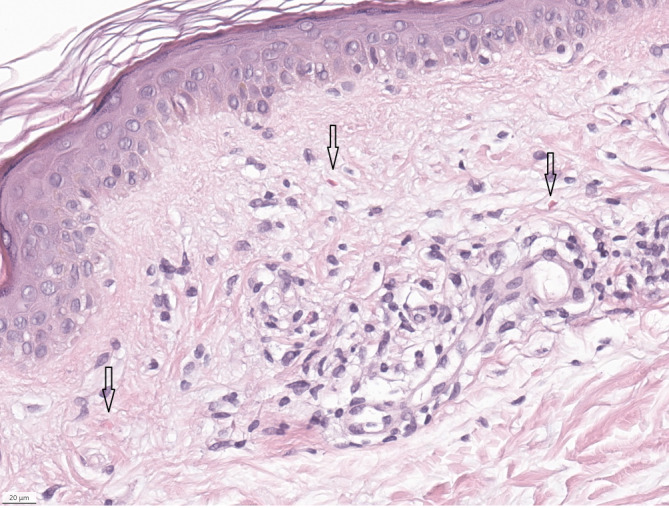
Ausschnittvergrößerung aus Abb. 3. Diskreter Nachweis extravasaler Erythrozyten im perivaskulären Infiltrat (*Pfeile*), passend zu einem frühen Stadium einer Purpura pigmentosa progressiva

Für andere Ursachen einer PPP, wie z. B. eine chronisch venöse Insuffizienz, zeigte sich klinisch und anamnestisch kein Hinweis.

Es wurde für eine Fortführung der Therapie mit Pembrolizumab entschieden. Nach der nächsten Gabe kam es zu keiner weiteren Befundzunahme. Die Behandlung wurde anschließend jedoch aufgrund des Auftretens einer Lungenmetastase beendet. Bei einer telefonischen Verlaufskontrolle 2 Monate nach der Vorstellung berichtete die Patientin, dass die Läsionen heller und insgesamt rückläufig seien – ohne spezifische dermatologische Therapie.

## Diskussion

Die PPP zählt zu den pigmentierten purpurischen Dermatosen und tritt meist idiopathisch auf, wurde jedoch auch im Zusammenhang mit Medikamenten beschrieben [1, 2]. Die exakte Pathophysiologie ist nicht vollständig geklärt, diskutiert werden T‑Zell-vermittelte Immunreaktionen auf kapilläre Endothelzellen.

Der Zusammenhang mit der Gabe von Pembrolizumab und dem Auftreten der PPP kann nicht final gesichert werden, der zeitliche Ablauf legt dies jedoch nahe. Im Kontext von Immuncheckpointinhibition sind kutane Nebenwirkungen häufig; typischerweise treten Exantheme, lichenoide Dermatosen oder Pruritus auf [3, 4]. In der Literatur wurde bisher ein einzelner Fall von PPP unter Pembrolizumab publiziert [2]. Die vorliegende Kasuistik unterstützt somit weiter die Hypothese, dass eine PPP als seltene Nebenwirkung von Immuncheckpointinhibitoren auftreten kann.
